# Integrating clinical proxies and metabolic data identifies and distinguishes high-risk depression subtypes in a real-world first-hospitalization cohort

**DOI:** 10.3389/fpsyt.2026.1798404

**Published:** 2026-03-17

**Authors:** Huizeng Yang, Pinfan Gu, Di Liu, Xinxu Wang, Minghui Li, Xinyu Xu, Nannan Liu

**Affiliations:** 1 Department of Psychiatry, Tianjin Anding Hospital, Mental Health Center of Tianjin Medical University, Tianjin, China; 2 School of Biomedical Engineering and Technology, Tianjin Medical University, Tianjin, China; 3 Department of Information Technology, Tianjin Anding Hospital, Mental Health Center of Tianjin Medical University, Tianjin, China; 4 Institute of Mental Health, Tianjin Anding Hospital, Mental Health Center of Tianjin Medical University, Tianjin, China; 5 Brain Assessment & Intervention Laboratory, Tianjin Anding Hospital, Mental Health Center of Tianjin Medical University, Tianjin, China

**Keywords:** clinical proxies, depressive disorder, first hospitalization, metabolic biomarkers, real-world data

## Abstract

**Background:**

Early identification of high-risk depression subtypes, specifically, recurrent (RD) and treatment-resistant (TRD) depression, is critical for improving long-term outcomes, yet practical stratification tools based on routinely available clinical and metabolic data remain limited. This study aimed to characterize these subtypes within a real-world, first-hospitalization cohort by integrating clinical proxy indicators with metabolic biomarkers.

**Methods:**

In a cross-sectional analysis of 1,436 first-hospitalized patients with first-episode depression (FED) and RD, we compared demographic, clinical, and metabolic characteristics. TRD was operationally defined by electroconvulsive therapy (ECT) exposure. Multivariable logistic regression identified factors associated with RD (vs. FED) and TRD (within RD).

**Results:**

Compared to FED patients, RD patients were older (47.1 vs. 42.4 years, p<0.001), had longer hospital stays, and exhibited a worse metabolic profile, including higher triglycerides (1.53 vs. 1.39 mmol/L, *p* = 0.014) and greater prevalence of elevated glucose (23.4% vs. 19.0%, p=0.047) and low HDL-C (39.5% vs. 31.4%, p=0.002). A disease duration >12 months was the strongest factor associated with a first-episode hospitalization diagnosis (OR = 10.75, p<0.001). Among RD patients, those with TRD (n=112) were distinguished by a higher rate of completing a 6-week treatment observation period (82.1% vs. 59.1%, p<0.001) and a greater prevalence of documented suicide risk (25.9% vs. 13.1%, *p* < 0.001). Completion of the observation period was the strongest predictor of TRD status (OR = 3.04, *p* < 0.001).

**Conclusion:**

In first-hospitalized patients, RD is associated with adverse metabolic markers, while TRD is characterized by clinical indicators of failed adequate treatment and high acute risk. A prolonged illness duration in first-episode patients may signal significant treatment delay. An integrated assessment of these accessible clinical and metabolic proxies could facilitate early risk stratification in routine care.

## 1 Introduction

Depressive disorders affect approximately 280 million people worldwide (1), representing a leading cause of years lived with disability and imposing a persistent burden on individuals, families, and healthcare systems (2, 3). While many patients achieve remission after initial treatment, the long-term course is often marked by chronicity and heterogeneity (4). A significant subset experiences relapse, known as recurrent depression (RD), and another portion demonstrates insufficient response to multiple treatments, meeting criteria for treatment-resistant depression (TRD) (5, 6). Compared to first-episode (FED) or treatment-responsive depression, RD and TRD are associated with greater symptom severity, functional impairment, and healthcare utilization (7). These high-risk subtypes necessitate early identification and tailored interventions, yet their empirical characterization remains fragmented, and practical stratification tools for routine care are limited.

Previous research has explored various biological and psychosocial correlates of depression recurrence and treatment resistance. However, findings regarding readily available clinical and routine laboratory parameters as predictors are inconsistent and lack integration (8). Specifically, the roles of metabolic dysregulation and immuno-metabolic pathways—increasingly implicated in depression severity and persistence -in differentiating RD from FED or distinguishing TRD are not well established (9, 10). Furthermore, while clinical indicators such as illness chronicity and prior treatment engagement are recognized (11, 12), their relative importance within a comprehensive framework that combines such clinical proxies with objective metabolic biomarkers is unclear.

A key limitation in the current literature is the scarcity of systematic, head-to-head comparisons of RD and TRD profiles using real-world data, especially from first-hospitalization cohorts. Such cohorts minimize confounding by prior extensive treatment (13, 14). Moreover, studies rarely integrate pragmatic clinical proxy indicators (e.g., structured observation periods, hospitalization length (8), treatment intensity) with metabolic biomarkers derived from standard blood tests (15). This integrated approach is crucial for developing practical, evidence-based tools for early risk stratification in clinical settings.

Therefore, leveraging a real-world, first-hospitalization cohort, this study aimed to: 1) systematically compare demographic, clinical, and metabolic characteristics between FED and RD patients; 2) identify the distinct profile of TRD (operationally defined by electroconvulsive therapy (ECT) exposure) versus non-TRD within the RD subgroup; and 3) determine independent factors associated with FED/RD and TRD/non-TRD status through multivariate modeling integrating clinical proxies and metabolic data. We hypothesized that RD and TRD would exhibit distinct patterns across these accessible metrics, with illness chronicity, specific metabolic parameters, and treatment engagement proxies emerging as significant associated factors. The findings aim to contribute to a more nuanced clinical profiling of high-risk depression subtypes, informing the development of practical stratification tools.

## 2 Methods

### 2.1 Data source

This study used data from the Electronic Health Records (EHR) system of Tianjin Anding Hospital. As previously described (16), a standardized data extraction and processing protocol was followed. This protocol involved using structured queries for coded data, applying natural language processing (NLP) to clinical text, and validating a sample of extracted data against the original records to ensure accuracy and build a reliable dataset (17, 18).

### 2.2 Study design and patient selection

We conducted a retrospective, cross-sectional analysis using real-world data from patients who had their first hospitalization at Tianjin Anding Hospital between January 1, 2013, and December 31, 2019. Patients were included if they: (1) were aged 18 to 65 years at admission; (2) were having their first recorded hospitalization with no prior outpatient or medication history in the system; and (3) had a primary discharge diagnosis of a depressive disorder (ICD-10 codes F32.x for single episode or F33.x for recurrent depression).

Patients were excluded for: (1) a history or current diagnosis of a major somatic disease; (2) pregnancy or lactation; or (3) a co-existing primary diagnosis of another major psychiatric disorder (e.g., schizophrenia, bipolar disorder). After applying these criteria, the final sample consisted of 1,436 patients.

### 2.3 Data extraction and variable definitions

We extracted demographic, clinical, and laboratory data from the EHR for each patient’s index hospitalization. Demographic and clinical variables included sex, age, ethnicity, marital status, family history of mental illness, and records of smoking and alcohol use.

Clinical proxy indicators, used in place of standardized severity scales, were defined as follows: Having a documented 6-week observation period was used as a proxy for determining whether a patient had received a standard, adequate initial treatment course. ECT exposure during the index hospitalization served as the operational criterion for identifying TRD, indicating insufficient response to prior treatment (19, 20). Duration of the current episode represented illness chronicity and was categorized as <3, 3-6, 6-12, or >12 months. The length of the initial and total hospitalization served as indicators of treatment intensity. Any documented suicide ideation, plan or attempt was recorded as a suicide risk behavior. The presence of documented non-suicidal self-injury (NSSI) was considered a distinct proxy variable reflecting maladaptive self-processing and dysregulated emotional coping (21, 22).

Laboratory metabolic indicators were taken from the first fasting blood test after admission. Indicators with over 30% missing data were excluded. The analyzed measures were: C-reactive protein (CRP), glucose, total cholesterol, triglycerides, high-density lipoprotein cholesterol (HDL-C), low-density lipoprotein cholesterol (LDL-C), and thyroid-stimulating hormone (TSH), total T3, and total T4.

A metabolic syndrome score (range 0-3) was calculated for each patient based on a modified application of the Joint Interim Statement (JIS) criteria (23). One point was assigned for each of the following present: (1) elevated triglycerides (≥1.7 mmol/L); (2) low HDL-C (<1.0 mmol/L in male, <1.3 mmol/L in female); and (3) elevated fasting glucose (≥5.6 mmol/L). Higher scores indicate greater metabolic abnormality.

### 2.4 Statistical analysis

All statistical analyses were performed using SPSS software (version 23.0). A two-sided p-value of less than 0.05 was considered statistically significant. Continuous variables are presented as mean ± standard deviation (SD), and categorical variables are summarized as frequencies and percentages. Prior to analysis, missing data were handled using multiple imputation via the Random Forest method, under the assumption that data were missing at random. To assess the robustness of this approach, a complete-case sensitivity analysis was performed (**Supplementary Table 1**). The Kolmogorov-Smirnov one-sample test was used to assess the normality of distribution for continuous variables. Group differences (FED vs. RD) in demographic and clinical characteristics, as well as in laboratory measures, were examined using the independent Student’s t-test for normally distributed continuous variables and the chi-square test for categorical variables. When more than 20% of cells in a contingency table had an expected count below 5, Fisher’s exact test was applied (24, 25).

Partial correlation analysis was conducted to evaluate the associations between blood physiological and biochemical parameters and other clinical variables, after adjusting for potential confounding factors. To identify factors associated with relapse risk and treatment resistance, forward stepwise logistic regression analysis was performed, incorporating symptom-related, sociodemographic, clinical, and medication-related variables. Candidate variables included all demographic, clinical, and metabolic parameters detailed in the Data Extraction section. Variables were entered using a score test p-value ≤ 0.05 and removed if the likelihood-ratio test p-value was ≥ 0.10. Multicollinearity was assessed using variance inflation factors (VIF); all retained variables had VIF < 2.0. The metabolic syndrome score was not entered simultaneously with its individual components (triglycerides, glucose, HDL-C) to avoid redundancy. Model calibration was evaluated with the Hosmer-Lemeshow goodness-of-fit test. The predictive performance of each logistic regression model was assessed by calculating the area under the receiver operating characteristic (ROC) curve (26, 27).

## 3 Results

### 3.1 Demographic and clinical characteristics of the participants

A total of 727 patients with FED and 709 with RD were included. The demographic and clinical characteristics of the two groups are summarized in **Table 1**. Patients with RD were significantly older than those with FED (47.13 ± 12.49 vs. 42.42 ± 13.78 years, *t* = −6.80, *p* < 0.001) and had a higher proportion of females (65.0% vs. 59.3%, χ² = 4.78, *p* = 0.029). No significant differences were observed in ethnicity, family history of mental illness, suicide risk, self-harm behavior, smoking, or alcohol use (all *p* > 0.05).

**Table 1:** Demographic, clinical and metabolic characteristics in FED and RD groups.

Variable	FED (N = 727)	RD (N = 709)	Statistics(t/X^2^)	*P-*value
Age	42.42 ± 13.78	47.13 ± 12.49	t=-6.80	**<0.001**
Sex			χ²=4.78	0.029
Female	431 (59.3%)	461 (65.0%)		
Man	296 (40.7%)	248 (35.0%)		
Ethnicity				0.917
Han	711 (97.8%)	697 (98.3%)		
Hui	7 (1.0%)	5 (0.7%)		
Manchu	2 (0.3%)	2 (0.3%)		
Other	7 (1.0%)	5 (0.7%)		
Family history			χ²=0.79	0.374
No	509 (70.0%)	480 (67.7%)		
Yes	218 (30.0%)	229 (32.3%)		
Total hospitalization days	58.32 ± 94.87	96.32 ± 191.49	t=-4.75	**<0.001**
First hospitalization days	42.01 ± 33.00	46.54 ± 32.54	t=-2.62	**0.009**
6-week observation period			χ²=36.42	<0.001
No	387 (53.2%)	264 (37.2%)		
Yes	340 (46.8%)	445 (62.8%)		
Disease duration			χ²=337.63	<0.001
3–6 months	132 (18.2%)	13 (1.8%)		
6–12 months	130 (17.9%)	30 (4.2%)		
>12 months	330 (45.4%)	642 (90.6%)		
<3 months	135 (18.6%)	24 (3.4%)		
Electroconvulsive therapy			χ²=12.39	<0.001
No	658 (90.5%)	597 (84.2%)		
Yes	69 (9.5%)	112 (15.8%)		
Suicide risk			χ²=1.32	0.251
No	600 (82.5%)	602 (84.9%)		
Yes	127 (17.5%)	107 (15.1%)		
Self-harm			χ²=0.03	0.854
No	717 (98.6%)	701 (98.9%)		
Yes	10 (1.4%)	8 (1.1%)		
Smoking			χ²=0.41	0.521
No	622 (85.6%)	597 (84.2%)		
Yes	105 (14.4%)	112 (15.8%)		
Alcohol use			χ²=3.15	0.076
No	699 (96.1%)	694 (97.9%)		
Yes	28 (3.9%)	15 (2.1%)		
CRP	2.84 ± 8.68	2.78 ± 8.12	t=0.13	0.899
Total cholesterol	4.90 ± 1.07	4.92 ± 1.04	t=-0.40	0.69
Glucose	5.21 ± 1.23	5.28 ± 1.14	t=-1.01	0.311
Triglycerides	1.39 ± 0.95	1.53 ± 1.14	t=-2.47	**0.014**
HDL-C	1.34 ± 0.29	1.32 ± 0.35	t=0.70	0.482
LDL-C	2.99 ± 0.97	2.99 ± 0.87	t=-0.03	0.973
TSH	2.40 ± 5.18	2.23 ± 1.54	t=0.87	0.386
T3	1.66 ± 0.38	1.68 ± 0.52	t=-0.78	0.438
T4	97.07 ± 19.09	98.43 ± 20.77	t=-1.30	0.195
Glucose score			χ²=3.96	0.047
<5.6	589 (81.0%)	543 (76.6%)		
≥5.6	138 (19.0%)	166 (23.4%)		
Triglycerides score			χ²=1.82	0.177
<1.7	528 (72.6%)	491 (69.3%)		
≥1.7	199 (27.4%)	218 (30.7%)		
HDL score			χ²=10.03	0.002
Low	228 (31.4%)	280 (39.5%)		
Normal	499 (68.6%)	429 (60.5%)		
Metabolic score			χ²=12.94	0.005
0	323 (44.4%)	266 (37.5%)		
1	262 (36.0%)	257 (36.2%)		
2	123 (16.9%)	151 (21.3%)		
3	19 (2.6%)	35 (4.9%)		

Bold means *p* < 0.05. FED, first episode depression. RD, recurrent depression. CRP, C-reactive protein. HDL-C, high-density lipoprotein cholesterol. LDL-C, low-density lipoprotein cholesterol. TSH, Thyroid-stimulating hormone. T3, Triiodothyronine, table shows total T3. T4, thyroxine, table shows total T4.

Clinically, RD patients had significantly longer total hospitalization days (96.32 ± 191.49 vs. 58.32 ± 94.87 days, *t* = −4.75, *p* < 0.001) and longer first hospitalization stays (46.54 ± 32.54 vs. 42.01 ± 33.00 days, *t* = −2.62, *p* = 0.009). The majority of RD patients had a disease duration exceeding 12 months (90.6% vs. 45.4% in FED, χ² = 337.63, *p* < 0.001). RD patients were also more likely to have undergone ECT (15.8% vs. 9.5%, χ² = 12.39, *p* < 0.001) and to have completed the 6-week observation period (62.8% vs. 46.8%, χ² = 36.42, *p* < 0.001).

### 3.2 Metabolic profile differences between patients with first-episode and recurrent depression

As also shown in **Table 1**, RD patients exhibited a less favorable metabolic profile compared to FED patients. Triglyceride levels were significantly higher in the RD group (1.53 ± 1.14 vs. 1.39 ± 0.95 mmol/L, *t* = −2.47, *p* = 0.014). Categorical analysis based on clinical cutoffs indicated that RD patients had a higher prevalence of elevated glucose (≥5.6 mmol/L; 23.4% vs. 19.0%, χ² = 3.96, *p* = 0.047) and low HDL-C (39.5% vs. 31.4%, χ² = 10.03, *p* = 0.002). The metabolic syndrome score distribution also differed significantly between groups (χ² = 12.94, *p* = 0.005), with RD patients more frequently in higher score categories. No significant differences were found for CRP, total cholesterol, fasting glucose (continuous), LDL-C, TSH, T3, or T4 (all *p* > 0.05).

### 3.3 Comparison between treatment-resistant and non-treatment-resistant depression patients

Among the RD subgroup, 112 patients (15.8%) who received ECT during the index hospitalization were classified as TRD. The remaining 597 RD patients (84.2%) constituted the non-TRD group. The demographic and clinical characteristics of these two subgroups are summarized in **Table 2**.

**Table 2:** Demographic, clinical and metabolic characteristics in TRD and non-TRD subgroups.

Variable	TRD (N = 112)	Non-TRD (N = 597)	Statistics(t/X^2^)	*P-*value
Age	49.90 ± 11.24	46.61 ± 12.65	t=2.78	**0.006**
Sex			χ²=2.08	0.149
Female	32 (28.6%)	216 (36.2%)		
Male	80 (71.4%)	381 (63.8%)		
Ethnicity			NA	0.875
Han	111 (99.1%)	586 (98.2%)		
Hui	0 (0.0%)	5 (0.8%)		
Manchu	0 (0.0%)	2 (0.3%)		
Other	1 (0.9%)	4 (0.7%)		
Family History			χ²=2.60	0.107
No	68 (60.7%)	412 (69.0%)		
Yes	44 (39.3%)	185 (31.0%)		
Total hospitalization days	147.95 ± 348.16	86.63 ± 142.80	t=1.84	0.069
First hospitalization days	56.80 ± 30.10	44.62 ± 32.64	t=3.88	**<0.001**
6-week observation period			χ²=20.40	<0.001
No	20 (17.9%)	244 (40.9%)		
Yes	92 (82.1%)	353 (59.1%)		
Disease duration			NA	0.395
<3 months	1 (0.9%)	23 (3.9%)		
3–6 months	2 (1.8%)	11 (1.8%)		
6–12 months	6 (5.4%)	24 (4.0%)		
>12 months	103 (92.0%)	539 (90.3%)		
Suicide Risk			χ²=11.13	<0.001
No	83 (74.1%)	519 (86.9%)		
Yes	29 (25.9%)	78 (13.1%)		
Self-harm			NA	0.368
No	110 (98.2%)	591 (99.0%)		
Yes	2 (1.8%)	6 (1.0%)		
Smoking			χ²=1.40	0.237
No	99 (88.4%)	498 (83.4%)		
Yes	13 (11.6%)	99 (16.6%)		
Alcohol use			NA	0.487
No	111 (99.1%)	583 (97.7%)		
Yes	1 (0.9%)	14 (2.3%)		
CRP	3.05 ± 5.25	2.73 ± 8.56	t=0.52	0.603
Total cholesterol	4.99 ± 1.05	4.91 ± 1.04	t=0.79	0.432
Glucose	5.44 ± 1.36	5.24 ± 1.10	t=1.45	0.148
Triglycerides	1.46 ± 0.86	1.54 ± 1.19	t=-0.84	0.402
HDL-C	1.32 ± 0.37	1.32 ± 0.34	t=0.14	0.887
LDL-C	3.07 ± 0.82	2.97 ± 0.88	t=1.09	0.278
TSH	2.10 ± 1.30	2.26 ± 1.57	t=-1.12	0.263
T3	1.62 ± 0.30	1.69 ± 0.56	t=-2.09	**0.037**
T4	99.66 ± 18.10	98.20 ± 21.24	t=0.76	0.447
Glucose score			χ²=1.65	0.199
<5.6	80 (71.4%)	463 (77.6%)		
≥5.6	32 (28.6%)	134 (22.4%)		
Triglycerides score			χ²=0.43	0.512
<1.7	81 (72.3%)	410 (68.7%)		
≥1.7	31 (27.7%)	187 (31.3%)		
HDL score			χ²=0.81	0.369
Low	63 (56.2%)	366 (61.3%)		
Normal	49 (43.8%)	231 (38.7%)		
Metabolic score			χ²=0.79	0.852
0	38 (33.9%)	228 (38.2%)		
1	42 (37.5%)	215 (36.0%)		
2	26 (23.2%)	125 (20.9%)		
3	6 (5.4%)	29 (4.9%)		

Bold means p < 0.05. FED, first episode depression. RD, recurrent depression. TRD, treatment-resistant depression. Non-TRD, non–treatment-resistant depression. CRP, C-reactive protein. HDL-C, high-density lipoprotein cholesterol. LDL-C, low-density lipoprotein cholesterol. TSH, Thyroid-stimulating hormone. T3, Triiodothyronine, table shows total T3. T4, thyroxine, table shows total T4.

TRD patients were significantly older than non-TRD patients (49.90 ± 11.24 vs. 46.61 ± 12.65 years, *t* = 2.78, *p* = 0.006). While no significant differences were observed in sex distribution, ethnicity, or family history of mental illness (all *p* > 0.05), notable clinical distinctions emerged. TRD patients had a significantly longer duration of their first hospitalization (56.80 ± 30.10 vs. 44.62 ± 32.64 days, *t* = 3.88, *p* < 0.001) and were substantially more likely to have completed the 6-week observation period (82.1% vs. 59.1%, χ² = 20.40, *p* < 0.001). Furthermore, TRD patients exhibited a significantly higher prevalence of documented suicide risk (25.9% vs. 13.1%, χ² = 11.13, *p* < 0.001).

Regarding metabolic and biochemical parameters, most measures, including lipid profiles, glucose, CRP, and thyroid function markers (TSH, T4), showed no significant inter-group differences (all *p* > 0.05). However, serum T3 levels were significantly lower in the TRD group in univariable analysis (1.62 ± 0.30 vs. 1.69 ± 0.56 nmol/L, *t* = -2.09, *p* = 0.037). This association did not retain statistical significance after multivariable adjustment (OR = 0.564, 95% CI: 0.273–1.166, p = 0.122). Categorical analyses of metabolic indicators revealed no statistically significant differences between TRD and non-TRD patients (all *p* > 0.05).

### 3.4 Factors associated with a first-episode depression presentation

To identify characteristics distinguishing patients hospitalized for a first depressive episode from those with recurrent episodes, we performed a stepwise multivariable logistic regression analysis with FED as the outcome (**Table 3**).

**Table 3:** Multivariable logistic regression analysis for factors associated with FED.

Variable	B	S.E.	Wald	df	*p*	Exp(B)	95% CI for Exp(B)	
							Lower	Upper
Constant	-3.267	0.327	100.126	1	**<0.001**	0.038	0.020	0.072
Disease duration (Ref: <3 months)								
3–6 months	-0.571	0.370	2.380	1	0.123	0.565	0.273	1.167
6–12 months	0.211	0.307	0.472	1	0.492	1.235	0.677	2.252
>12 months	2.379	0.236	101.304	1	**<0.001**	10.75	6.80	16.98
Age (per year)	0.022	0.005	21.455	1	**<0.001**	1.022	1.013	1.032
6-week obs: Yes	0.578	0.131	19.642	1	**<0.001**	1.782	1.381	2.300
Female	0.229	0.133	2.965	1	0.085	1.257	0.969	1.631
Alcohol: Yes	-0.766	0.376	4.147	1	**0.042**	0.465	0.222	0.972
HDL-C: Low	0.262	0.133	3.910	1	**0.048**	1.300	1.002	1.686
ECT: Yes	0.316	0.195	2.622	1	0.105	1.372	0.935	2.008

Bold means *p* < 0.05. The dependent variable is First-Episode Depression (FED = 1, RD = 0). An Odds Ratio (Exp(B)) greater than 1 indicates the variable or category is associated with higher odds of being in the FED group. An OR less than 1 indicates association with lower odds of FED (i.e., higher odds of RD). 6-week obs: Yes, patients completed a 6-week structured observation period. Alcohol: Yes, history of alcohol use. HDL-C, high-density lipoprotein cholesterol. HDL: Low, low HDL-C <1.0 mmol/L in men, <1.3 mmol/L in women. ECT, electroconvulsive therapy. ETC Yes, has undergone ECT.

A disease duration exceeding 12 months prior to index hospitalization was the strongest factor associated with a first-episode presentation. Compared to patients with a very recent onset (<3 months), those with an episode lasting over 12 months had more than tenfold higher odds of being classified as FED (OR = 10.75, 95% CI: 6.80–16.98, *p* < 0.001). Conversely, older age was associated with a reduced likelihood of FED classification, with each additional year linked to approximately 2% higher odds of being in the RD group (OR for FED = 1.022 per year, 95% CI: 1.013–1.032, *p* < 0.001).

Completion of a 6-week observation period, a proxy for adequate treatment trial, was also negatively associated with a first-episode diagnosis. Patients who completed this period had 78% higher odds of belonging to the RD group (OR for FED = 1.782, 95% CI: 1.381–2.300, *p* < 0.001). A history of alcohol use was a distinctive marker for recurrence, associated with 53% lower odds of a first-episode presentation (OR = 0.465, 95% CI: 0.222–0.972, *p* = 0.042). Furthermore, having a low HDL-C level was associated with a 30% higher odds of being in the FED group (OR = 1.300, 95% CI: 1.002–1.686, *p* = 0.048). Female sex and a history of ECT did not show independent associations in the final model (both *p* > 0.05).

### 3.5 Factors associated with treatment-resistant depression (ECT exposure) in recurrent depression

Within the RD cohort, we examined factors associated with receiving ECT, our operational criterion for TRD. A stepwise multivariable logistic regression was conducted with ECT exposure as the outcome (**Table 4**). The analysis identified two robust clinical correlates. Completion of the 6-week observation period was the strongest predictor, associated with a three-fold increase in the odds of receiving ECT (OR = 3.037, 95% CI: 1.810–5.096, *p* < 0.001). The presence of documented suicidal risk at assessment also significantly increased the odds of ECT exposure twofold (OR = 2.213, 95% CI: 1.342–3.648, *p* = 0.002). Lower levels of serum total T3 (OR = 0.564, *p* = 0.122) and older age (OR = 1.016 per year, *p* = 0.089) were included in the final model but did not reach statistical significance.

**Table 4:** Stepwise multivariable logistic regression analysis of factors associated with TRD (ECT exposure) in RD.

Variable	B	S.E.	Wald	df	*p*	Exp(B)	95% CI for Exp(B)	
							Lower	Upper
Constant	-2.44	0.79	9.539	1	**0.002**	0.087	0.019	0.41
6-week obs: Yes	1.111	0.264	17.707	1	**<0.001**	3.037	1.81	5.096
Suicide: Yes	0.794	0.255	9.694	1	**0.002**	2.213	1.342	3.648
Total T3	-0.573	0.37	2.391	1	0.122	0.564	0.273	1.166
Age	0.016	0.009	2.895	1	0.089	1.016	0.998	1.035

Bold means *p* < 0.05. The dependent variable is electroconvulsive therapy (ECT) exposure during the index hospitalization, which served as the operational definition for treatment-resistant depression (TRD) in this study. An Odds Ratio (Exp(B)) greater than 1 indicates the factor is associated with higher odds of receiving ECT (i.e., TRD). 6-week obs, 6-week observation. 6-week obs: Yes, patients completed a 6-week structured observation period. Suicide: Yes, have any documented suicide attempt. T3, Triiodothyronine.

### 3.6 Regression models and discriminative performance

**Figure 1:**
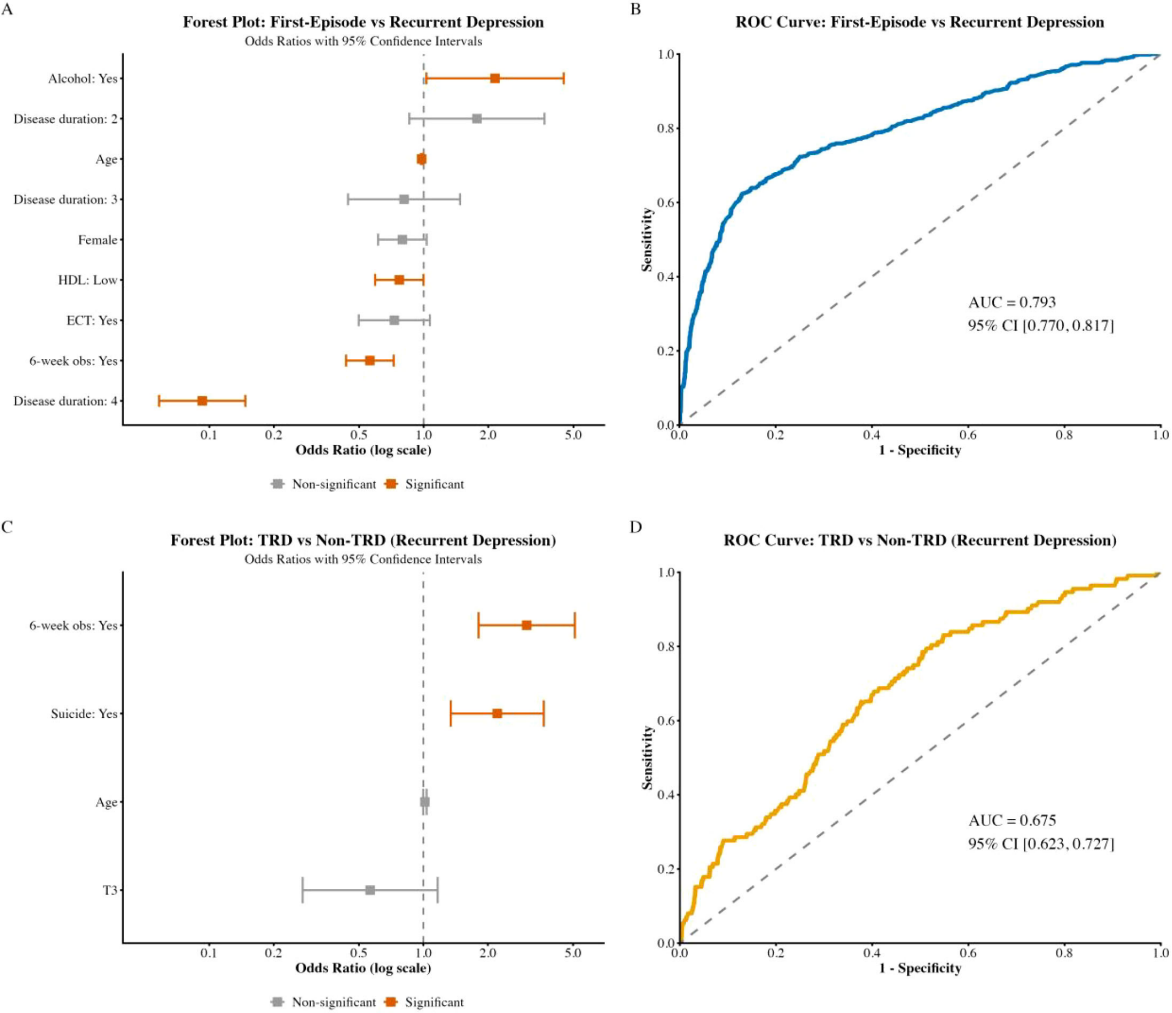
Multivariable logistic regression model summaries and performance. **(A)** Forest plot showing odds ratios for factors associated with first-episode versus recurrent depression. Orange markers indicate statistically significant factors (p < 0.05), gray markers indicate non-significant variables. **(B)** ROC curve for the model distinguishing first-episode from recurrent depression (AUC = 0.793, 95% CI 0.770–0.817). **(C)** Forest plot showing odds ratios for factors associated with treatment-resistant depression versus non-treatment-resistant depression within the recurrent depression subgroup. **(D)** ROC curve for the model distinguishing treatment-resistant from non-treatment-resistant depression (AUC = 0.675, 95% CI 0.623–0.727).

**Figure 1** shows that in the stepwise multivariable logistic regression, alcohol use, age, low HDL-C, and completion of a 6-week observation period emerged as statistically significant correlates distinguishing FED from RD (**Figure 1A**). In addition, a longer disease-duration category (disease duration was over 12 months marked as 4 in **Figure 1A**) showed a significant association, whereas other duration strata (e.g., 3–6 and 6–12 months, marked as 2 and 3 respectively), female sex, and ECT history were not retained as significant predictors (**Figure 1A**). Discrimination of the final model was with an AUC of 0.793 (95% CI 0.770–0.817) (**Figure 1B**). Within the RD subgroup, stepwise multivariable logistic regression identified the 6-week observation period and suicidality as significant predictors of TRD status, while age and T3 did not reach statistical significance (**Figure 1C**). The corresponding ROC analysis showed moderate discriminatory performance, with an AUC of 0.675 (95% CI 0.623–0.727) (**Figure 1D**).

## 4 Discussion

In this real-world cohort of first-hospitalized patients with depression, we identified distinct profiles for RD and TRD subtypes by integrating clinical proxies with metabolic data. Compared to FED patients, those with RD were older, had longer hospital stays, more often completed a 6-week treatment observation period, and exhibited a worse metabolic profile, including higher triglycerides and a greater prevalence of dysglycemia and low HDL-C.

Within the RD subgroup, patients with TRD (operationally defined by ECT exposure) were primarily distinguished by clinical trajectory rather than metabolic markers. They were significantly more likely to have completed the 6-week observation period and to have documented suicide risk.

Multivariable modeling revealed that a disease duration exceeding 12 months was the strongest factor associated with a first-episode hospitalization diagnosis. For TRD, completion of the 6-week observation period and the presence of suicidality were the key associated clinical factors.

### 4.1 Metabolic dysregulation and illness course

The observed association between a less favorable metabolic profile characterized by elevated triglycerides, dysglycemia, and low HDL-C and RD aligns with the growing immuno-metabolic depression framework, which posits a bidirectional link between metabolic dysfunction and depression chronicity (9). Our findings are consistent with prior reports of a higher prevalence of metabolic syndrome in recurrent compared to first-episode cases (28) and extend this association to a carefully characterized first-hospitalization cohort, suggesting that metabolic disturbances are linked to the recurrent phenotype independent of long-term treatment exposure (29).

Potential mechanisms underlying this association may involve inflammation- and glucocorticoid-mediated impairments in neuroplasticity, including reduced brain-derived neurotrophic factor (BDNF) signaling, as well as a persistently dysregulated hypothalamic-pituitary-adrenal (HPA) axis (30, 31), which together may foster a cycle of relapse and metabolic compromise (32). Supporting the relevance of peripheral biomarkers even in early-stage illness, previous work has shown that metabolic-inflammatory markers such as albumin can predict rehospitalization risk in first-episode patients (33), echoing our finding that metabolic disturbances are already more pronounced in first-hospitalized RD patients.

Thus, our results provide real-world clinical support for the role of metabolic health in depression course, highlighting routine metabolic measures as potential markers for stratifying recurrence risk early in the illness trajectory.

### 4.2 Possible treatment delay as an explanatory factor

A key and non-intuitive finding in our study was that a disease duration exceeding 12 months was the strongest factor linked to a first-episode hospitalization diagnosis. This observation warrants careful consideration of two distinct, yet not mutually exclusive, interpretations. First, it may reflect substantial treatment delay prior to admission, wherein the index hospitalization represents not the true onset of illness but the endpoint of prolonged unrecognized or inadequately managed symptoms. This interpretation aligns with the established literature on duration of untreated depression (DUD) (34), which is associated with poorer treatment outcomes and increased recurrence risk (35, 36).

Second, this association could instead signify an intrinsically insidious disease onset, characterized by gradual accumulation of subthreshold symptoms that only later cross the diagnostic threshold, possibly representing a distinct neurobiological subtype (37, 38). Clinically, both scenarios mandate enhanced vigilance: treatment delay points to health-system interventions (e.g., improving detection and access), whereas insidious onset suggests closer monitoring of patients with prolonged prodromal phases, even absent prior treatment history. In either case, a long pre-admission illness duration should not be dismissed as benign, but rather prompt careful r-evaluation of illness trajectory and consideration of more intensive follow-up.

### 4.3 Clinical proxies for treatment resistance

In this study, TRD was operationally defined by ECT exposure during hospitalization, a pragmatic marker of insufficient response to standard care in this real-world cohort (39, 40). While clinically clear, this definition may not capture all resistant cases and can be influenced by local treatment practices (20).

Notably, completion of the 6-week observation period was the strongest predictor of ECT use, which is consistent with previous research (41, 42). This association highlights a key TRD pathway: patients who complete an adequate initial treatment trial yet remain severely ill enough to require ECT. Thus, this period serves as a valid proxy for identifying probable non-response.

Furthermore, documented suicidal risk was significantly associated with TRD status, consistent with established literature linking greater illness severity and suicidality with treatment resistance (43, 44). Together, these clinically accessible indicators help profile TRD within RD. It should be noted, however, that the discriminative performance of the TRD prediction model was modest (AUC = 0.675), indicating that these clinical proxies alone are not sufficiently accurate for standalone decision-making. This finding provides a realistic benchmark and highlights the need for future studies to incorporate additional predictors, such as longitudinal treatment response data or novel biomarkers, to improve predictive accuracy.

### 4.4 Implications for integrated assessment and targeted management

This study demonstrates that integrating easily obtainable clinical proxies—such as illness duration, completion of a structured treatment observation period, and hospitalization length—with routine metabolic blood tests can effectively stratify high-risk depression subtypes. This pragmatic, multi-domain framework is particularly suitable for real-world clinical settings with limited resources, aiding in the early identification of patients with RD or those at potential risk for TRD who may benefit from closer monitoring and intensified intervention.

By way of illustration, one metabolic parameter, low HDL-C, may serve as a readily available signal of recurrence risk. In our multivariable analysis, low HDL-C was independently associated with RD (OR = 1.30, p = 0.048). This lipid measure is already routinely collected on standard admission panels; therefore, at no additional cost, a low HDL-C value could prompt clinicians to reassess the possibility of prior depressive episodes and consider closer longitudinal monitoring, even in patients presenting with an apparent first hospitalization.

Our findings suggest several targeted clinical actions. For patients with RD, routine screening for metabolic syndrome and the integration of lifestyle interventions into treatment plans are warranted. For patients hospitalized with a “first episode” but with a notably long illness duration, clinicians should proactively assess for a history of treatment delay or initial poor response, rather than assuming a typical first-onset course. Furthermore, the completion of an adequate treatment trial (e.g., the 6-week period) in the presence of high suicidal risk should alert clinicians to a significantly elevated likelihood of TRD, prompting consideration of earlier and more aggressive treatment strategies.

### 4.5 Limitations

Several limitations of this study should be acknowledged. First, the cross-sectional design precludes inferences regarding causality. Second, the single-center design may limit the generalizability of our findings. This is particularly relevant for the metabolic parameters examined, which are known to vary across populations due to differences in dietary habits, lifestyle behaviors (e.g., physical activity, smoking, alcohol consumption), and genetic background. While the direction of the associations observed in this study is likely to be consistent across settings, the magnitude of effect sizes may differ. Multicenter studies incorporating diverse geographic and demographic populations are therefore required to establish the robustness and external validity of these metabolic signatures. Third, the reliance on clinical proxy indicators (e.g., the 6-week observation period, ECT exposure for TRD) rather than standardized assessment tools, while pragmatic, may introduce measurement bias. Fourth, potential confounding factors such as detailed medication regimens, psychotherapy, and levels of social support were not fully captured. Although we excluded patients with prior outpatient medication records, the influence of antidepressant use on metabolic parameters cannot be entirely ruled out, as undocumented prior treatment or in-hospital medication changes may have contributed to the observed metabolic differences. Finally, metabolic indicators were measured only once at admission and do not reflect their dynamic changes over time.

## 5 Conclusion

In this real-world, first-hospitalization cohort, recurrent depression was linked to adverse metabolic profiles. A prolonged illness duration was strongly associated with a first-episode diagnosis, suggesting a clinically significant subgroup with probable treatment delay. Additionally, failure to respond to an adequate treatment trial and high suicidal risk were key identifiers of treatment resistance. Future prospective studies are needed to validate these markers and to explore targeted interventions for these high-risk profiles.

## Data Availability

The original contributions presented in the study are included in the article/**Supplementary Material**. Further inquiries can be directed to the corresponding author/s.
