# Supplementary material for: Integrating clinical proxies and metabolic data identifies and distinguishes high-risk depression subtypes in a real-world first-hospitalization cohort
Source: Front Psychiatry. 2026 Mar 17;17:1798404. doi: 10.3389/fpsyt.2026.1798404 (PMC13035803; doi:10.3389/fpsyt.2026.1798404)
Supplement: Supplementary file 1 [file Table1.docx]

Table S1 Comparison of Primary Analysis and Sensitivity Analysis

1. FED vs RD Comparison

| Variable | Primary OR (95% CI) | p | Sensitivity OR (95% CI) | p | Consistent |
| --- | --- | --- | --- | --- | --- |
| Disease duration ( > 12 months) | 14.76 (9.35-23.29) | <0.001 | 13.32 (7.85-22.61) | <0.001 | Yes |
| Age | 1.02 (1.01-1.03) | <0.001 | 1.03 (1.02-1.04) | <0.001 | Yes |
| 6-week observation period (Yes) | 1.78 (1.38-2.30) | <0.001 | 1.48 (1.11-1.97) | 0.008 | Yes |
| Gender (Female) | 1.26 (0.97-1.63) | 0.085 | 1.48 (1.11-1.98) | 0.008 | Yes* |
| Alcohol use (Yes) | 0.46 (0.22-0.97) | 0.042 | 0.44 (0.20-0.99) | 0.047 | Yes |
| HDL score (Low) | 1.30 (1.00-1.69) | 0.048 | — | — | — |
| Electroconvulsive therapy (Yes) | 1.37 (0.94-2.01) | 0.105 | 1.50 (0.98-2.28) | 0.060 | Yes |

**Sample size**: Primary N=1,436 (imputed); Sensitivity N=1,153 (complete case)

**Model performance**: Primary AUC=0.793 (0.770-0.817); Sensitivity AUC=0.803 (0.777-0.828)

1. TRD vs Non-TRD Comparison

| Variable | Primary OR (95% CI) | p | Sensitivity OR (95% CI) | p | Consistent |
| --- | --- | --- | --- | --- | --- |
| 6-week observation period (Yes) | 3.04 (1.81-5.10) | <0.001 | 3.01 (1.78-5.08) | <0.001 | Yes |
| Suicide attempt (Yes) | 2.21 (1.34-3.65) | 0.002 | 2.52 (1.50-4.25) | <0.001 | Yes |
| T3 | 0.56 (0.27-1.17) | 0.122 | 0.61 (0.30-1.24) | 0.172 | Yes |
| Age | 1.02 (1.00-1.04) | 0.089 | — | — | — |

**Sample size**: Primary N=709 (imputed); Sensitivity N=599 (complete case)

**Model performance**: Primary AUC=0.675 (0.623-0.727); Sensitivity AUC=0.672 (0.614-0.730)
